# Prokineticin 1 modulates IL-8 expression via the calcineurin/NFAT signaling pathway

**DOI:** 10.1016/j.bbamcr.2009.03.008

**Published:** 2009-07

**Authors:** David Maldonado-Pérez, Pamela Brown, Kevin Morgan, Robert P. Millar, E. Aubrey Thompson, Henry N. Jabbour

**Affiliations:** a Human Reproductive Sciences Unit, Medical Research Council, Edinburgh EH16 4TJ, UK; b Department of Cancer Biology, Mayo Clinic Comprehensive Cancer Center, Jacksonville, Florida 32224, USA

**Keywords:** Prokineticin 1, IL-8, RCAN1-4, NFAT, Endometrium

## Abstract

Prokineticins and their receptors are expressed in various cellular compartments in human endometrium, with prokineticin 1 (PROK1) showing a dynamic pattern of expression across the menstrual cycle and during pregnancy. Previous studies suggest that PROK1 can play an important role in implantation and early pregnancy by inducing vascular remodeling and increasing vascular permeability. Here we demonstrate that PROK1 induces the expression of IL-8, a chemokine with angiogenic properties, in endometrial epithelial Ishikawa cells stably expressing prokineticin receptor 1 and in human first trimester decidua. We also show that IL-8 promoter activity is induced by PROK1 and that this requires the presence of AP1 and NFAT motifs. The role of calcineurin/NFAT signaling pathway is confirmed by the use of specific chemical inhibitors. Additionally, PROK1 induces the expression of the regulator of calcineurin 1 isoform 4 (RCAN1-4) via the calcineurin/NFAT pathway. A modulatory role for RCAN1-4 is demonstrated by RCAN1-4 overexpression which results in the inhibition of PROK1-induced IL-8 expression whereas reduction in RCAN1-4 endogenous expression results in an increase in PROK1-induced IL-8 production. Our findings show that in endometrial cells PROK1 can activate the calcineurin/NFAT pathway to induce IL-8 expression and that this is negatively modulated by the induction of expression of RCAN1-4.

## 1 Introduction

The prokineticins, prokineticin 1 (PROK1) and prokineticin 2 (PROK2), are two secreted factors that have been described to play roles in multiple processes such as intestinal contraction [1], vascular function [2], hematopoiesis [3,4] and the development of the olfactory and GnRH systems [5,6]. These effects are induced via binding to and activation of their cognate receptors, two G protein-coupled receptors called prokineticin receptor 1 (PROKR1) and prokineticin receptor 2 (PROKR2). Both receptors bind to PROK1 and PROK2 with similar affinities [7,8] and studies in vitro have shown they can couple to Gi and Gq proteins [2,7,8] and activate intracellular signaling molecules such as PLC, ERK, Akt and eNOS [2,7,9,10].

Prokineticins and their receptors are widely expressed in both male and female reproductive systems [11]. In the uterus, the expression of prokineticins and their receptors has been described with only PROK1 showing a temporal pattern of expression across the menstrual cycle; with maximal levels of expression observed during the mid secretory phase [12,13]. PROK1 has been localized to endometrial glandular and luminal epithelium, stromal and endothelial cells as well as myometrial vascular endothelium and smooth muscle [12,13]. In addition, expression of PROK1 and PROKR1 is elevated in first trimester decidualized endometrium compared to non-pregnant endometrium [10]. This pattern of expression suggests that PROK1 could have an important role in the physiology of the non-pregnant and pregnant endometrium. The central role that angiogenesis plays in endometrial physiology together with the angiogenic properties reported for prokineticins lead us to investigate the possible role of prokineticins in the induction of expression of angiogenic factors. IL-8 is a factor that belongs to the CXC chemokine family, whose known actions include neutrophil chemotactic/activating and T-cell chemotactic activity [14], as well as chemotaxis and proliferation of endothelial cells in vitro and angiogenesis in vivo [15]. Previous studies have demonstrated that PROK1 induced the expression of IL-8 in monocytes [16] and third trimester placenta [17]. In the present study we investigated the role of PROK1 in the modulation of IL-8 expression and secretion. We confirmed that activation of PROKR1 by PROK1 in human endometrial epithelial cells resulted in the induction of expression of IL-8 and we demonstrated that this induction occurred via the activation of the calcineurin/nuclear factor of activated T-cells (NFAT) signaling pathway. We also showed that PROK1 via the same pathway induced the expression of the regulator of calcineurin 1 isoform 4 (RCAN1-4), an endogenous modulator of the calcineurin signaling pathway. Expression of RCAN1-4 negatively modulated the expression of IL-8 induced by PROK1.

## 2 Materials and methods

### 2.1 Reagents

DMEM nutrient mixture F-12 culture medium was purchased from Invitrogen (Paisley, UK). YM-254890 was kindly donated by Astellas Pharma Inc (Tsukuba, Japan). NF-κB SN50 cell permeable inhibitory peptide was purchased from Biomol International (Exeter, UK). Cyclosporin A and Inhibitor of NFAT-Calcineurin Association-6 (Inca-6) were purchased from Calbiochem (Nottingham, UK). Flag-tagged NFATc1 plasmid construct was a kind gift from Dr Tania N. Crotti (Beth Israel Deaconess Medical Center, Boston, MA).

### 2.2 Cell culture

Ishikawa endometrial adenocarcinoma cells were obtained from the European Collection of Cell Culture (Wiltshire, UK). Stable PROKR1 transfectant cells were designed and characterized as described before [10]. These cells were cultured in DMEM/F-12 cultured medium supplemented with 10% fetal bovine serum and a maintenance dose of 200 μg/ml of G418 antibiotic.

### 2.3 Tissue collection

First trimester decidua (9–12 wk, *n* = 7) was collected from women undergoing elective first trimester surgical termination of pregnancy. Ethical approval was obtained from Lothian Local Research Ethics Committee, and written informed consent obtained from all patients before tissue collection.

### 2.4 Angiogenesis array

The human angiogenesis antibody array I (RayBiotech, Inc., Norcross, GA) was used according to the manufacturer's instructions. Briefly, the membranes were incubated with 2 ml blocking buffer for 30 min, followed by 2 h incubation with 1 ml of conditioned medium collected from cultured Ishikawa PROKR1 cells treated with or without 40 nM PROK1 for 8 h. After washing, the membranes were incubated overnight with 1 ml of biotin-conjugated antibodies solution. The membranes were then washed, incubated with 1 ml HRP-conjugated streptavidin for 2 h and washed. Subsequently the membranes were incubated in detection buffer mix for 2 min and exposed to X-ray film for 5–30 s. Densitometric analysis was performed using the ImageQuant TL software (GE Healthcare, Little Chalfont, UK).

### 2.5 Taqman quantitative RT-PCR

Total RNA was extracted from cells and tissues using Total RNA Isolation Reagent (Abgene, Epsom, UK). Quantified RNA samples were reverse transcribed and quantitative RT-PCR was performed as described before [18] using the following primers and probes: RCAN1: forward: 5′-CGCCAAATCCAGACAAGCA-3′; reverse: 5′-CGCATCTTCCACTTGTTTCCA-3′ and probe: 5′-FAM-TCTCCC CTCCCGCCTCTCCG-3′; RCAN1-4: forward: 5′-GAAAGTGAAACCAGGGCCAAA-3′; reverse: 5′-GCTGGAGCCTGGCATCTG-3′ and probe: 5′-FAM-TCAGAATAAACTTCAGCAACCCCTTCTCCG-3′; IL-8: forward: 5′-CTGGCCGTGGCTCTCTTG-3′; reverse: 5′-TTAGCACTCCTTGGCAAAACTG-3′ and probe: 5′-FAM-CCTTCCTGATTTCTGCAGCTCTGTGTGAA-3′. Custom primers and probes for RCAN, calcineurin and NFAT different isoforms were purchased from Applied Biosystems (Foster City, CA). The expression of analyzed genes was normalized for RNA loading using 18S rRNA or GAPDH as internal standards.

### 2.6 Immunohistochemistry

Tissue section dual immunofluorescent histochemistry was carried out as previously described [17], using a goat anti-IL-8 antibody (1:20; R&D Systems Europe Ltd., Abingdon, UK) and a rabbit anti-PROKR1 antibody (1:100; MBL, Woburn, MA).

### 2.7 Western blot analysis

Protein concentration of cell lysates was quantified using Bio-Rad protein assay kit (Bio-Rad Laboratories, Hemel Hempstead, UK). After resolving and blotting, membranes were incubated overnight at 4 °C, with a rabbit anti RCAN-1 antibody (1:5000), a kind gift from Dr Erik W. Bush (Myogen, Inc, Westminster, CO), together with a mouse anti β-actin antibody (1:800) (Santa Cruz Biotechnology, Santa Cruz, CA). The following day, cells were washed and incubated with goat anti-rabbit Alexafluor 680 (1:5000; Invitrogen) and goat anti-mouse IRDye™ 800 (1:5000; Rockland, Gilbersville, PA) for 60 min at room temperature. Blots were visualized and the protein immunoreactivity quantified using an Odyssey infrared imaging system (LI-COR, Cambridge, UK). RCAN1 relative density was calculated by dividing the value obtained for RCAN1 by the value obtained for β-actin and expressed as fold above vehicle controls.

### 2.8 Secreted IL-8 quantification

Secreted IL-8 was quantified using an in-house enzyme linked immunosorbent assay (ELISA) described previously [19]. A matched pair of capture and biotinylated labeled detection antibodies for IL-8 and recombinant IL-8 were used (R&D Systems, Oxford, UK).

### 2.9 Luciferase reporter assay

pNF-κB-Luc and pAP1-Luc vectors were purchased from Clontech (Mountain View, CA). RCAN1-4 promoter reporter plasmids and NFAT_4_-Luc were kindly donated by Dr Takashi Minami (University of Tokyo, Tokyo, Japan) [20]. The IL-8 promoter reporter constructs were kindly donated by Dr Allan R. Brasier (Department of Internal Medicine, University of Texas Medical Branch, Galveston, Texas) [21].

Ishikawa PROKR1 cells were plated in 24 well plates at a density of 60,000 cells/well. After 24 h of incubation, cells were co-transfected with one of the different promoter reporter plasmids used in combination with a *Renilla* luciferase internal control vector pRL-TK (Promega, Southampton, UK; 10:1 promoter reporter plasmid:pRL-TK) using Superfect transfection reagent (QIAGEN, Crawley, UK) following manufacturer's guidelines. The following day the cells were serum starved for 16 h. Cells were then treated in serum free media. After this, cells were lysed and the activity of both firefly and *Renilla* luciferase on each sample was determined using the dual luciferase assay kit (Promega).

### 2.10 RCAN1-4 adenovirus infection

The cDNA of RCAN1-4 (ORIGENE, Rockville, MD) was excised with EcoRI and SmaI and fused to EcoRI and SmaI restricted pDC316 shuttle vector (Microbix Biopharmaceuticals, Toronto, Canada) to create pDC316-RCAN1-4.

HEK 293 cells (ATCC CRL 1573) were cultured in MEM + Glutamax medium (Invitrogen) containing 10% FCS and 1% Penicillin/Streptomycin. Cells were transfected with 0.5 μg pDC316-RCAN1-4 and 1.5 μg adenoviral genomic plasmid pBHGloxΔ E1,3 Cre (Microbix) using TransIT-293 as per manufacturer's instructions (Mirus Bio Corp, Madison, WI). Adenoviral plaques were harvested 10–14 days later and virus released by 3 × freeze/thaw cycles. Clonal plaques were obtained by serial dilution and infection of 80% confluent HEK 293 cells overlaid 5 h post inoculation with 0.5% SeaPlaque Agarose (FMC Corp, Rockland, ME) dissolved in growth media. Plaques were picked 8–12 days later, inoculated into a T75 flask and incubated until 70%–80% cytopathic effect (CPE) was observed. This first seed was inoculated into multiple flasks and harvested when CPE was apparent. RCAN1-4 Adenovirus was purified, concentrated, aliquoted and stored at − 80 °C (Vivapure AdenoPACK 100 purification kit; Sartorius AG, Goettingen, Germany). Titers were determined using the AdenoX Rapid titer kit (CloneTech). Yields of in excess of 1 × 10^10^ IFU/ml were routinely obtained.

Ishikawa PROKR1 cells were plated in 6 well plates at a density of 200,000 cells/well. After 24 h of incubation, cells were washed with PBS and 1 ml of fresh medium containing 5 adenovirus pfu/plated cell was added to each well. Cells were incubated for another 24 h and serum starved overnight before treatment with 40 nM PROK1.

### 2.11 Lentivirus shRNA gene silencing

A short hairpin RNA (shRNA) lentivirus, previously described [22], was used to knock down the expression of RCAN1. Briefly, Ishikawa PROKR1 cells were plated in 12 well plates at a density of 80,000 cells/well. After 24 h of incubation, cells were infected with virus-containing media at a 1:10 dilution of virus to target cell media and 0.6 μg/ml Polybrene. The day after, medium was replaced with fresh serum-containing medium and 48 h post-infection, the cells which were serum starved overnight, were treated with 40 nM PROK1.

### 2.12 Statistical analysis

The data in this study was analyzed by *t* test, ANOVA or Kruskal–Wallis nonparametric test using Prism 4.0c (Graph Pad, San Diego, CA).

## 3 Results

### 3.1 PROK1 induces the expression of IL-8 in human endometrial Ishikawa cells and first trimester decidua

**Fig. 1:**
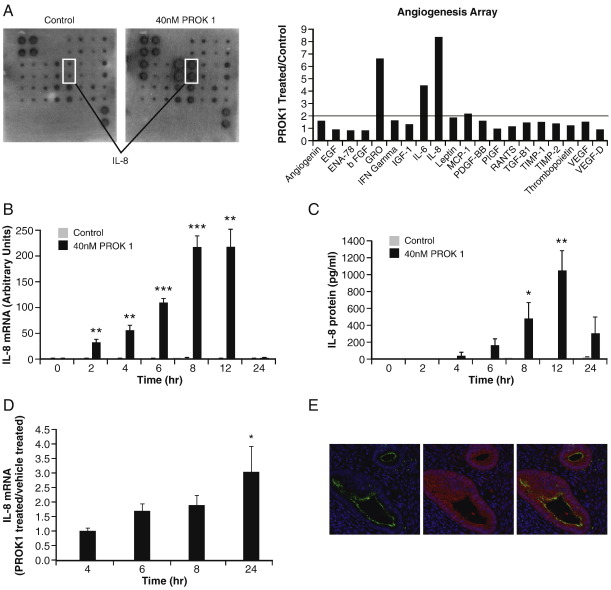
PROK1 induces the expression of IL-8 in Ishikawa PROKR1 cells and human first trimester decidua. (A) Conditioned medium from Ishikawa PROKR1 cells treated with vehicle or 40 nM PROK1 for 8 h was tested for the expression of angiogenic factors using the human angiogenesis antibody array I. Expression of GRO, IL-6, IL-8 and MCP-1 was upregulated by more than 2 fold by treatment with PROK1. (B) Ishikawa PROKR1 cells treated with 40 nM PROK1 showed a significant increase in the expression of IL-8 mRNA but not control treated cells. (C) Similarly, IL-8 protein secretion was significantly increased by 40 nM PROK1 treatment. (D) Human first trimester decidua explants treated with 40 nM PROK1 showed a significant increase in the expression of IL-8 mRNA at 24 h. (E) Flouresent immuno histochemistry showed that PROKR1 (red) and IL-8 (green) co-immunolocalized (yellow) in glandular epithelium of first trimester human decidua. Data in B, C, and D are presented as mean ± S.E. of *n* = 3–7 experiments ⁎*p* < 0.05, ⁎⁎*p* < 0.01, ⁎⁎⁎*p* < 0.001. Control = vehicle treatment of Ishikawa PROKR1 cells.

In order to investigate the potential role of PROK1 on the induction of angiogenic factors in endometrial cells, we made use of a human endometrial adenocarcinoma Ishikawa cells [23], stably expressing PROKR1 [10]. Conditioned medium collected from cells treated with 40 nM PROK1 or vehicle for 8 h was used in an angiogenesis protein array. The array showed that the chemokines: GRO, IL-6, IL-8 and MCP-1 were upregulated by more than two-fold following treatment with PROK1, with IL-8 showing the highest upregulation (> 8 fold increase) (Fig. 1A).

We decided to investigate in more detail the induction of IL-8 by PROK1 based on documented expression of this chemokine in the non-pregnant and pregnant uterus [24] and its known roles in angiogenesis [15]. A previous study in our group showed the induction of IL-8 mRNA by PROK1 in Ishikawa PROKR1 cells by gene array analysis [10]. In agreement with these previous results, time courses of IL-8 mRNA levels (Fig. 1B) and IL-8 protein secretion (Fig. 1C) in response to treatment with 40 nM PROK1 confirmed that PROK1 treatment increased IL-8 mRNA and protein (*p* < 0.01). Similarly, Ishikawa PROKR1 cells transfected with a construct coding for the promoter region of IL-8 linked to the luciferase reporter gene [21] and treated with 40 nM PROK1 showed a significant induction of IL-8 promoter activity (Supplementary Fig. 1).

We also tested the induction of IL-8 by PROK1 in first trimester decidua explants. Treatment with 40 nM PROK1 induced a significant increase in IL-8 expression (*p* < 0.05; Fig. 1D). In addition, dual fluorescent immunohistochemical analysis showed that PROKR1 and IL-8 are co-expressed in the epithelium of first trimester decidua (Fig. 1E).

### 3.2 PROK1 induces activation of IL-8 promoter through AP1 and NFAT

**Fig. 2:**
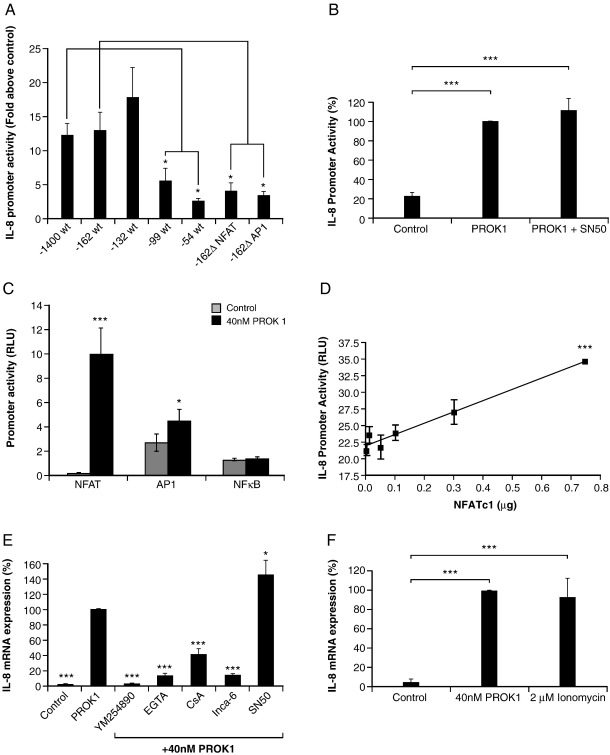
PROK1 induces IL-8 via NFAT and AP1 but not NF-κB. (A) The use of truncated versions of the wild type (wt) IL-8 promoter-luciferase construct allowed the identification of the region between − 132 nucleotides and − 54 nucleotides to be important for activation by treatment with PROK1. Furthermore, the use of − 162 truncated constructs containing point mutations identified AP1 and NFAT binding sites to be required for IL-8 promoter activation. Data are presented as fold increase of PROK1 treated cells (6 h) above control treated cells. (B) PROK1-induced IL-8 promoter activity at 6 h was not inhibited by an NF-κB inhibitory peptide (SN50, 100 μg/ml). Data are presented as percentage of RLU relative to PROK1 treated cells. (C) The use of luciferase constructs containing specific binding sites for NFAT, NF-κB and AP1 show that PROK1 treatment for 6 h induced activation of the NFAT and AP1 constructs but not NF-κB. (D) PROK1-induced IL-8 promoter activity was increased by transiently transfecting Ishikawa PROKR1 cells with an NFATc1 plasmid. The increase in promoter activity induced by NFATc1 plasmid was dose dependent. (E) The induction of IL-8 mRNA by PROK1 at 6 h could be significantly inhibited by the use of Gαq/11 inhibitor (YM-254890, 100 nM), calcium chelator (EGTA, 1.5 mM), calcineurin inhibitor (cyclosporine, 1 μM), and NFAT inhibitor (Inca-6, 40 μM). In contrast, a significant increase in IL-8 mRNA expression was observed when cells were pretreated with NF-κB inhibitory peptide (SN50, 100 μg/ml). Data are presented as percentage of mRNA expression relative to PROK1 treated cells. (F) Treatment of Ishikawa PROKR1 cells with an ionophore (ionomycin, 2 μM) for 2 h resulted in an increase in IL-8 mRNA similar to that induced by PROK1. Data are presented as percentage of mRNA expression relative to PROK1 treated cells. All data are presented as mean ± S.E. of *n* = 3–4 experiments. ⁎*p* < 0.05, ⁎⁎⁎*p* < 0.001. Control = vehicle treatment of Ishikawa PROKR1 cells.

To identify promoter regions involved in the induction of expression of IL-8 by PROK1, truncated versions of the IL-8 promoter construct were used. A considerable reduction in promoter activity was observed when the promoter was truncated down to − 99 nucleotides of the 5′ flanking region (*p* < 0.05). A further reduction in promoter activity was observed in the − 54 nucleotides truncated construct which only contains the IL-8 TATA box (*p* < 0.05; Fig. 2A). These results suggested that the region between − 132 and − 54 nucleotides was required for IL-8 gene activation by PROK1.

Site directed mutated versions of the − 162 IL-8 promoter at consensus binding sequences for AP-1 (− 125 to − 119 nucleotides) and NFAT (− 80 to − 75 nucleotides) showed that these binding sites are required for PROK1-induced transcription of IL-8 (Fig. 2A). The NFAT binding site in the IL-8 promoter is part of a κB-like consensus sequence that can also bind NF-κB. In order to determine if NF-κB was involved in the activation of IL-8 promoter in Ishikawa PROKR1 cells we made use of the NF-κB inhibitory peptide SN50 (100 μg/ml) which did not reduce the activity of IL-8 promoter induced by PROK1 (Fig. 2B). Also, Ishikawa PROKR1 cells transfected with the pNF-κB-Luc vector (Promega) and treated with 40 nM PROK1 did not induce NF-κB-driven luciferase activity. In contrast, luciferase reporter constructs containing NFAT or AP1 binding sites were readily activated by treatment with PROK1 (*p* < 0.05; Fig. 2C).

### 3.3 The calcineurin signaling pathway is involved in PROK1-induced expression of IL-8

Having identified that NFATc transcription factors may be important in IL-8 induction, we quantified the mRNA expression of NFATc isoforms in Ishikawa PROKR1 cells. Expression of NFATc1, NFATc2, NFATc3 and NFATc4 message was detected in these cells. Treatment with 40 nM PROK1 did not induce a significant change in the expression of NFATc isoforms (Supplementary Fig. 2).

We subsequently analysed the role of the calcineurin/NFATc pathway in IL-8 induction by PROK1. We co-transfected cells with an NFATc1 construct and the IL-8 promoter-luciferase construct into Ishikawa PROKR1 cells. Transfection of the NFATc1 construct induced an increase in IL-8 promoter activity in a dose dependent manner (Fig. 2D).

In addition, the use of chemical inhibitors confirmed the role of the calcineurin signaling pathway in the induction of IL-8 expression in Ishikawa PROKR1 cells. Pretreatment of Ishikawa PROKR1 cells with chemical inhibitors for Gq (YM254890), calcineurin (cyclosporine A) and NFAT (Inca-6), as well as an extracellular calcium chelator (EGTA) resulted in a significant inhibition of PROK1-induced expression of IL-8 mRNA (*p* < 0.001). These inhibitors did not significantly change the basal expression of IL-8 (data not shown). In contrast, the use of an NF-κB inhibitory peptide (SN50) induced an increase in the expression of IL-8 induced by PROK1 (Fig. 2E), and basal expression of IL-8 was also significantly induced by SN50 (data not shown). On the other hand, the use of an ionophore (ionomycin), which allows extracellular calcium entry to the cytoplasm, resulted in a similar induction of IL-8 mRNA to that induced by PROK1 giving further support to the role of the calcium-dependent calcineurin signaling pathway (Fig. 2F).

### 3.4 PROK1 induces the expression of the calcineurin negative modulator RCAN1

Having identified that the calcineurin signaling pathway is required for the induction of expression of IL-8 by PROK1, we then explored the induction by PROK1 of the regulator of calcineurin 1 (RCAN1), also known as Down syndrome critical region gene 1 (DSCR1) or Adapt 78 [25].

**Fig. 3:**
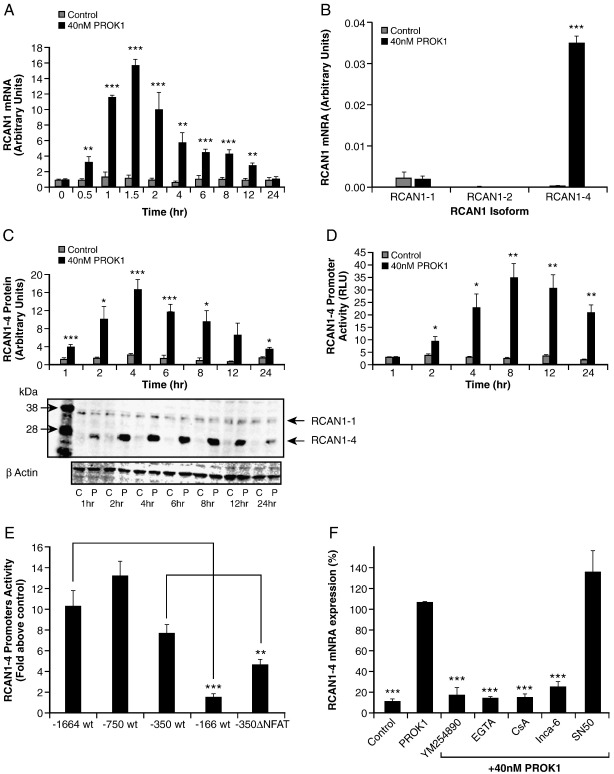
RCAN1-4 expression is induced by PROK1 via activation of the calcineurin/NFAT pathway. (A) Treatment of Ishikawa PROKR1 cells with 40 nM PROK1 resulted in a rapid increase in the expression of RCAN1 mRNA. (B) mRNA quantification of the different isoforms of RCAN-1 show that PROK1 treatment for 2 h specifically induced mRNA expression of isoform 4 of RCAN1. (C) Western blot analysis showed that protein levels of RCAN1 isoform 4 (28 kDa), but not isoform 1 (38 kDa), were upregulated by PROK1. The expression of RCAN1-4 was normalized using β-actin as a loading control. C = vehicle and P = PROK1 treated cells. (D) RCAN1-4 promoter-luciferase construct was activated by treatment of Ishikawa PROKR1 cells with 40 nM PROK1 but not by control treatment. (E) The use of truncated versions of the RCAN1-4 promoter-luciferase construct showed that the activity of RCAN1-4 was reduced when the promoter was truncated between − 350 and − 166 nucleotides. A point mutation on one of the NFAT binding sites remaining on the − 350 nucleotides truncated promoter (− 350ΔNFAT) resulted in a significant reduction in promoter activity. Data are presented as fold increase of PROK1 treated cells (6 h) above control treated cells. (F) The use of Gαq/11 inhibitor (YM-254890, 100 nM), calcium chelator (EGTA, 1.5 mM), calcineurin inhibitor (cyclosporine, 1 μM), and NFAT inhibitor (Inca-6, 40 μM) resulted in a significant inhibition of PROK1-induced expression of RCAN1-4 mRNA after 6 h treatment. In contrast, no reduction in RCAN1-4 mRNA expression was observed with NF-κB inhibitory peptide (SN50, 100 μg/ml). Data are presented as percentage of mRNA expression relative to PROK1 treated cells. All data are presented as mean ± S.E. of *n* = 3–5 experiments. ⁎*p* < 0.05, ⁎⁎*p* < 0.01, ⁎⁎⁎*p* < 0.001. Control = vehicle treatment of Ishikawa PROKR1 cells.

Treatment of Ishikawa PROKR1 cells with 40 nM PROK1 resulted in rapid induction of RCAN1 mRNA (*p* < 0.001; Fig. 3A). The use of RCAN1-4 isoform specific primers showed that PROK1 induces the expression of isoform 4 (*p* < 0.001) but not isoforms 1 nor 2 (Fig. 3B). RCAN1 isoform 4 (RCAN1-4) is an endogenous modulator of the calcineurin signaling pathway and previous studies have identified that PROK1 can induce expression of RCAN1 by gene array analysis [10]. Similarly, treatment of first trimester decidua with 40 nM PROK1 resulted in a significant increase in the expression of RCAN1-4 at 2 h (Supplementary Fig. 3).

Also, treatment of Ishikawa PROKR1 cells with 40 nM PROK1 resulted in the induction of expression of RCAN1-4 protein (28 kDa; *p* < 0.001) [26]. In contrast RCAN1 isoform 1 (38 kDa band) [26] expression was not increased by PROK1 treatment (Fig. 3C).

Making use of a promoter-luciferase construct, we observed that PROK1 induced the activity of RCAN1-4 promoter (*p* < 0.01; Fig. 3D). It has been reported that RCAN1-4 promoter region contains 15 NFAT binding sites [27]. To investigate the role of NFAT in the modulation of the expression of RCAN1-4 induced by treatment with PROK1, we made use of truncated versions of RCAN1-4 promoter. The results showed that PROK1-induced RCAN1-4 promoter activation was only significantly impaired when the promoter was truncated down to − 166 nucleotides (*p* < 0.001). This truncated version of RCAN1-4 promoter contains some NFATc putative binding sites but they were not sufficient for activation of gene transcription (Fig. 3E). We then made use of a − 350 nucleotides RCAN1-4 promoter construct containing a point mutation in one of the NFAT binding sites (− 350ΔNFAT) to test whether NFATc binding to this promoter is involved in the activation of RCAN1-4. This promoter construct showed a significantly reduced activity in comparison to − 350 wild type (*p* < 0.01; Fig. 3E).

The use of chemical inhibitors confirmed that the calcineurin/NFAT signaling pathway is involved in the induction of RCAN1-4. Inhibitors for Gq (YM254890), calcineurin (cyclosporine A) and NFAT (Inca-6) as well as an extracellular calcium chelator (EGTA) significantly inhibited the expression of RCAN1-4 mRNA (*p* < 0.001). However, an NF-κB inhibitory peptide (SN50) did not have an effect on RCAN1-4 expression (Fig. 3F). In contrast, the use of ionomycin resulted in a similar induction of RCAN1-4 mRNA to that induced by PROK1 (Supplementary Fig. 4).

### 3.5 RCAN1-4 overexpression inhibits PROK1-induced expression of IL-8

**Fig. 4:**
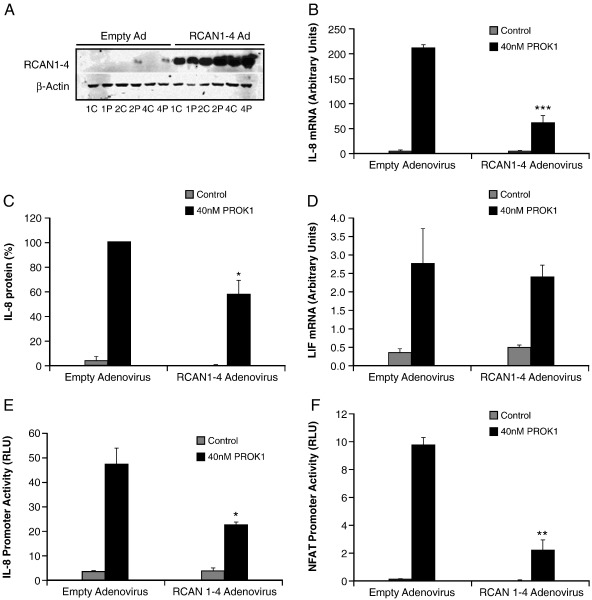
Overexpression of RCAN1-4 results in downregulation of PROK1-induced expression of IL-8. (A) Western blot analysis showed that Ishikawa PROKR1 cells infected with an RCAN1-4 adenovirus showed an increased expression of this protein compared to cells infected with a control empty adenovirus. Cells were treated with vehicle (C) or 40 nM PROK1 (P) for 1, 2 and 4 h. (B) Ishikawa PROKR1 cells overexpressing RCAN1-4 showed a significant reduction in the amount of IL-8 mRNA induced by treatment with 40 nM PROK1 for 8 h in comparison to cell infected with an empty adenovirus. (C) IL-8 protein secretion induced by PROK1 was significantly reduced in RCAN1-4 adenovirus infected cells in comparison to empty adenovirus infected cells. Data are presented as percentage of protein expression relative to empty adenovirus infected cells treated with PROK1 for 8 h. (D) RCAN1-4 overexpression did not affect PROK1-induced expression of LIF, a gene not known to be modulated by the calcineurin/NFAT pathway. (E) PROK1-induced activation of IL-8-luciferase promoter construct was also significantly reduced by overexpression of RCAN1-4 at 8 h. (F) Similarly, the activity of an NFAT-luciferase promoter construct at 6 h was significantly reduced when RCAN1-4 was overexpressed in Ishikawa PROKR1 cells. Data represent the mean ± S.E. of *n* = 4 experiments. ⁎*p* < 0.05, ⁎⁎*p* < 0.01, ⁎⁎⁎*p* < 0.001. Control = vehicle treatment of Ishikawa PROKR1 cells.

In order to investigate further the role of NFAT in the induction of expression of IL-8 and the role of RCAN1-4 in this process, we overexpressed RCAN1-4, which is known to bind to calcineurin and inhibit activation of NFATc when overexpressed [28]. For this we used an adenoviral construct coding for RCAN1-4 that induced an increase in expression of RCAN1-4 protein in infected Ishikawa PROKR1 cells (Fig. 4A).

RCAN1-4 overexpression resulted in a significant reduction in PROK1-induced expression of IL-8 mRNA in Ishikawa PROKR1 cells (*p* < 0.001; Fig. 4B). In addition, the amount of IL-8 protein secreted was also significantly reduced (*p* < 0.05; Fig. 4C). In order to determine if the observed inhibitory effect of overexpressing RCAN1-4 was specific, we looked at the effect of RCAN1-4 adenovirus on the expression of leukemia inhibitory factor (LIF), a gene known to be induced by PROK1 in Ishikawa PROKR1 cells [10] and that is not known to be modulated via NFAT. No significant reduction of PROK1-induced LIF mRNA expression was observed in cells infected with RCAN1-4 adenovirus when compared to cells infected with control adenovirus (Fig. 4D).

In agreement with the above results, the activity of IL-8 promoter in Ishikawa cells stimulated with PROK1 showed a significant reduction when RCAN1-4 was overexpressed (*p* < 0.05; Fig. 4E). The inhibitory effect of RCAN1-4 overexpression on activation of IL-8 promoter could be explained by the known role of RCAN1-4 as an inhibitor of NFAT activation [28]. In order to test this, the activity of the NFAT-luciferase promoter construct induced by treatment with PROK1 was measured in cells infected with empty or RCAN1-4 adenovirus. Overexpression of RCAN1-4 produced a significant reduction in the activation of NFAT by PROK1 (*p* < 0.01; Fig. 4F).

### 3.6 Inhibition of endogenous RCAN1-4 expression results in an increased expression of PROK1-induced IL-8

**Fig. 5:**
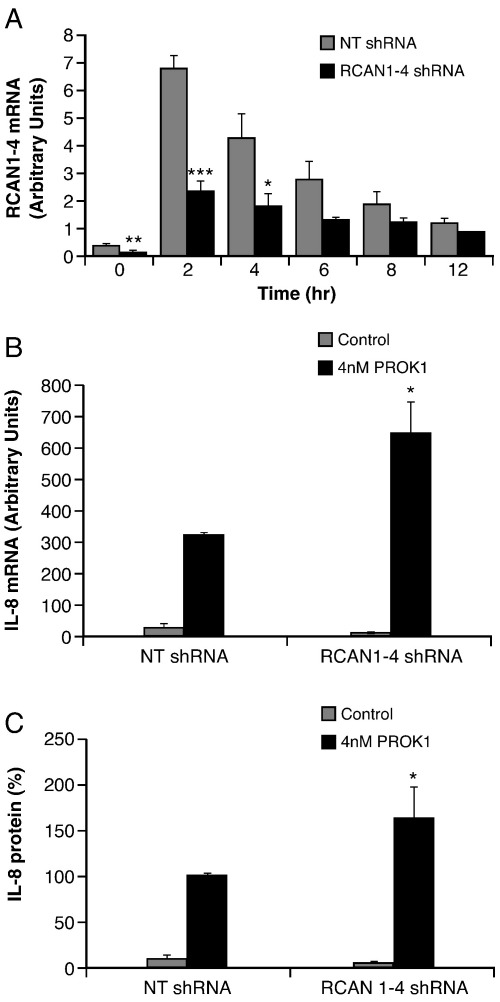
Inhibition of endogenous RCAN1-4 results in an increase in IL-8 induction by PROK1. (A) Ishikawa PROKR1 cells infected with a lentivirus expressing an shRNA to RCAN1 showed a significant reduction in the expression of endogenous RCAN1-4 mRNA compared to cells infected with a non target (NT) control lentivirus. (B) Reduction in the expression of endogenous RCAN1-4 resulted in a significant increase in the expression of IL-8 mRNA induced after treatment with 4 nM PROK1 for 12 h. (C) Secreted IL-8 protein was also higher in cells infected with RCAN1 shRNA lentivirus compared to NT control infected cells after treatment with 4 nM PROK1 for 12 h. Data are presented as percentage of protein expression relative to empty NTshRNA infected cells treated with PROK1. All data are presented as mean ± S.E. of *n* = 3–4 experiments. ⁎*p* < 0.05, ⁎⁎*p* < 0.01, ⁎⁎⁎*p* < 0.001. Control = vehicle treatment of Ishikawa PROKR1 cells.

In order to give further support to the role of RCAN1-4 as a negative modulator of PROK1-induced expression of IL-8, we made use of an shRNA lentiviral construct that targets RCAN1. Infection of Ishikawa PROKR1 cells with RCAN1 shRNA lentivirus resulted in a reduction in the expression of basal levels of endogenous RCAN1-4 mRNA (*p* < 0.01; Fig. 5A, time 0 h). In addition, PROK1-induced increase in RCAN1-4 mRNA expression was significantly reduced in RCAN1 shRNA infected cells compared to non target control (NT) shRNA infected cells (*p* < 0.001; Fig. 5A, time 2 h). On the other hand, infection of cells with RCAN1 shRNA resulted in an increase in the expression PROK1-induced IL-8 mRNA (*p* < 0.05; Fig. 5B) and protein (*p* < 0.05; Fig. 5C) compared to cells infected with NT shRNA.

## 4 Discussion

In the human endometrium, PROK1 expression is temporally regulated. Expression of this factor is elevated during the window of implantation and early pregnancy [10,12]. In the present study we show that PROK1 induces the expression of chemokines that are known to have angiogenic properties. We focused our attention on studying the expression of IL-8 due to its known importance in endometrial physiology. This chemokine is secreted by endometrial stromal and epithelial cells in culture [29] as well as in cultured early pregnancy decidua [30]. In vivo, IL-8 is expressed in glandular cells and surface epithelium of the endometrium [31], where this chemokine could be involved in several processes of endometrial physiology such as angiogenesis, proliferation, chemotaxis, trophoblast invasion and uterine contraction [24].

Our results show that PROK1-PROKR1 interaction results in a significant induction of IL-8 in Ishikawa PROKR1 cells and first trimester decidua. Treatment of Ishikawa PROKR1 cells with PROK1 induces a significant increase in IL-8 mRNA and protein secretion, and the activity of its promoter is significantly increased. The kinetics of IL-8 promoter activity is different from the mRNA expression kinetics. While IL-8 mRNA expression levels are down to basal levels by 24 h after treatments with PROK1 (Fig. 1D), the promoter activity is still elevated at this time point (Supplementary Fig. 1). We believe that this difference is explained by the fact that luciferase is a non-human gene that can be submitted to different post-translational regulation to that of endogenously expressed IL-8. Similarly, PROK1 induces a significant increase in the expression of mRNA in first trimester decidua where the PROKR1 and IL-8 co-localize to the epithelial compartment.

The induction of IL-8 expression by PROK1-PROKR1 interaction occurs via activation of the calcineurin signaling pathway. Although NF-κB has been shown to bind to the κB-like motif present in the IL-8 promoter and modulate its activity [32]; in some cases specific inhibition of NF-κB does not impair the production of IL-8 [33]. On the other hand, NFATc proteins have been shown to bind several sequences that resemble binding sites for Rel family proteins [34]. In fact, NFATc isoforms can bind to the κB-like motif in the IL-8 promoter [35] and can modulate IL-8 promoter activity [36,37]. In Ishikawa PROKR1 cells the modulation of IL-8 by PROK1 is independent of NF-κB but instead modulated by the calcineurin/NFATc signaling pathway. Interestingly, the use of an inhibitor of NF-κB resulted in a significant increase in the expression of IL-8. This suggests that in the absence of basal NF-κB nuclear localization, NFAT can access more readily the binding region in the IL-8 promoter which as mentioned above is also recognized by NF-κB.

IL-8 promoter induction by NFAT required the presence of the neighboring AP1 binding site, suggesting that both NFATc and AP1 transcription factors are necessary to activate the IL-8 promoter in endometrial epithelial cells. It has been previously shown that AP1 and NFAT can mutually stabilize each other's interaction with DNA binding domains of promoters of several genes to allow full gene transactivation to occur [38,39]. It is plausible that activation of IL-8 promoter occurs as a result of AP1 and NFAT interaction. These results highlight the fact that activation of IL-8 promoter appears to be differentially modulated by different transcription factor complexes and this appears to depend on the cellular context and/or the conditions of stimulation.

In agreement with the identification of RCAN1 as a target gene for PROK1 in endometrial cells by gene array analysis [10], we also show in the present study that PROK1 treatment induced the expression of RCAN1 mRNA and protein. Further analysis identified that PROK1 specifically induced the expression of RCAN1 isoform 4. The expression of this isoform is known to be induced by NFATc [20]. The use of truncations and point mutated versions of RCAN1-4 promoter together with the use of specific chemical inhibitors confirmed that the induction of RCAN1-4 by PROK1 occurs via the calcineurin/NFATc signaling pathway.

The role of the calcineurin/NFAT signaling pathway in the PROK1-induced expression of IL-8 was supported by the inhibitory effect observed when RCAN1-4 was overexpressed in our model system. Infection of Ishikawa PROKR1 cells with an adenoviral construct containing RCAN1-4 reduced the IL-8 mRNA expression in response to PROK1. RCAN1-4 is known to bind to calcineurin and previous studies have shown that overexpression of this protein results in an inhibition of calcineurin activation of NFAT [20,28]. These results also suggested that endogenous expression of RCAN1-4 induced by PROK1 could be playing a role as a negative modulator of the calcineurin/NFAT pathway. This is further supported by our observation that infection of Ishikawa PROKR1 cells with an RCAN1 shRNA lentivirus construct resulted in a significant reduction in the expression of RCAN1-4. This reduction in the expression of RCAN1-4 resulted in an augmented expression of PROK1-induced expression of IL-8.

**Fig. 6:**
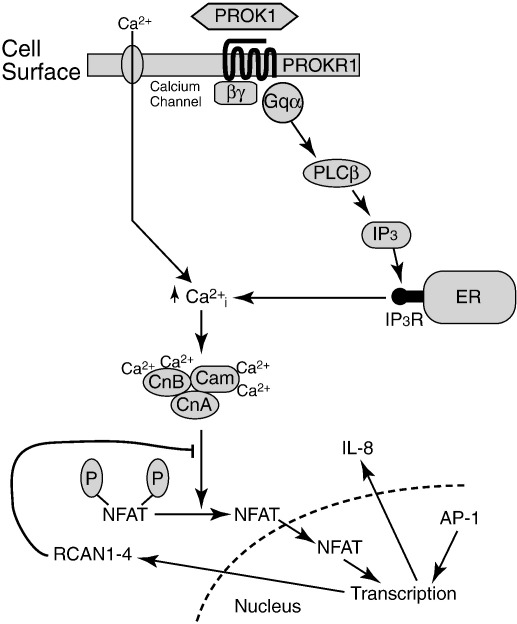
Schematic representation of PROK1 induction of RCAN1-4 and IL-8. Activation of the PROKR1 by PROK1 results in the induction of IL-8 and RCAN1-4 expression. This occurs via coupling of PROKR1 to Gq protein. Activation of PLCβ by Gqα results in the production of IP_3_ which can then bind to IP_3_R in the ER and result in an efflux of calcium from the ER into the cytoplasm. In addition, the opening of calcium channels in the cell surface can also contribute to the increase in Ca^2+^_i_. This increase in Ca^2+^_i_ results in calmodulin saturation, which in turn activates calcineurin. Activated calcineurin then dephosphorylates cytoplasmic NFAT. Dephosphorylated NFAT can then migrate to the nucleus and bind to binding motifs in the promoter region of RCAN1-4 and IL-8 and induce their transcription. NFAT-induced transcription of IL-8 occurs in conjunction with AP-1. The accumulation of RCAN1-4 protein results in its binding to calcineurin and the inhibition of NFAT dephosphorylation by calcineurin which prevents further expression of IL-8 and RCAN1-4. Gqα = Gq protein alpha subunit, βγ = G protein beta gamma subunits, PLCβ = phopholipase C beta, IP_3_ = inositol-1,4,5-triphosphate, IP_3_R = IP_3_ receptor, ER = endoplasmic reticulum, Ca^2+^_i_ = intracellular ionized calcium, Cam = calmodulin, CnB = calcineurin regulatory subunit, CnA = calcineurin catalytic subunit, NFAT = nuclear factor of activated T-cells, AP-1 = activator protein 1.

In summary, our results show that PROK1-PROKR1 interaction results in the activation of the calcineurin signaling pathway which in turn induces the expression of RCAN1-4 and, in combination with AP1 also induces the expression of IL-8. The accumulation of RCAN1-4 then results in the inhibition of the calcineurin signaling pathway which causes a reduction in RCAN1-4 and IL-8 expression (Fig. 6). It is important to remark that it has been previously demonstrated that RCAN1-4 can itself be subject to post-translational regulation. It has been shown that oxidative stress [40] and MAP kinase signaling [41] can induce phosphorylation of RCAN1-4. In addition, RCAN1-4 has been shown to stimulate the production of active GSK-3β which is known to be involved in phosphorylation of both RCAN1-4 as well as NFAT [42]. Phosphorylation of RCAN1-4 favors the dissociation of this protein from calcineurin as well as promoting its degradation whereas phosphorylation of NFAT results in the inactivation of this transcription factor. Therefore, it appears that the modulatory effect of RCAN1-4 on the calcineurin/NFAT signaling pathway is itself tightly regulated.

Spatial and temporal regulation of chemokine secretion in the endometrium is essential since these factors are involved in crucial processes of uterine physiology such as vascular remodeling, implantation and parturition. The involvement of PROK1 in vascular function has been previously demonstrated [43–45]. In addition, PROK2 via activation of PROKR1 promotes survival and angiogenesis in cardiomyocytes [46]. The identification of several angiogenic genes induced by PROK1 via activation of PROKR1 [10] together with the data presented here strongly support a role for PROK1-PROKR1 in vascular function in the endometrium. At the same time, previous studies in vitro have shown that RCAN1-4 is involved in the modulation of vascular function induced by the vascular endothelial growth factor in endothelial cells [47–49]. Our results suggest that PROK1-PROKR1 has a role in the modulation of angiogenic events in the endometrium in part via IL-8, and that this process could be modulated by the action of RCAN1-4. The modulation of IL-8 secretion by RCAN1-4 could have an important role in the adequate regulation of secreted IL-8 in the endometrium which would prevent undesirable effects by dysregulated spatial and/or temporal synthesis of IL-8. It is also possible that dysregulated expression of PROK1, PROKR1 or RCAN1-4 could be involved in pathologies of vascular function of the endometrium and in pregnancy. Further experiments in vivo are necessary to understand the exact role of PROK1 in vascular function in the female reproductive tract as well as the involvement of RCAN1-4 in these processes.

## Appendix A Supplementary data

Supplementary Fig. 1 An IL-8 promoter-luciferase construct transfected into Ishikawa PROKR1 cells was activated by treatment with 40 nM PROK1 but not by control treatment. Data are presented as mean ± S.E. of *n* = 3 experiments. ⁎*p* < 0.05, ⁎⁎*p* < 0.01, ⁎⁎⁎*p* < 0.001. Control = vehicle treatment of Ishikawa PROKR1 cells.

Supplementary Fig. 2 mRNA expression for NFAT isoforms NFATc1, NFATc2, NFATc3 and NFATc4 was detected in Ishikawa PROKR1 cells. No induction of NFATc isoforms was observed in response to treatment with 40 nM PROK1 for 8 h. Data are presented as mean ± S.E. of *n* = 3 experiments.

Supplementary Fig. 3 Human first trimester decidua treated with 40 nM PROK1 showed a significant increase in the expression of RCAN1-4 mRNA. Data are presented as mean ± S.E. of *n* = 7 experiments. ⁎*p* < 0.05.

Supplementary Fig. 4 Treatment of Ishikawa PROKR1 cells with ionomycin (2 μM) resulted in an increase in RCAN1-4 mRNA similar to that induced by PROK1. Data is presented as percentage of mRNA expression relative to PROK1 treated cells and represent the mean ± S.E. of *n* = 3 experiments. ⁎⁎⁎*p* < 0.001. Control = vehicle treatment of Ishikawa PROKR1 cells.
