# Supplementary figures and images for: Prokineticin 1 modulates IL-8 expression via the calcineurin/NFAT signaling pathway
Source: Biochim Biophys Acta. 2009 Jul;1793(7):1315–24. doi: 10.1016/j.bbamcr.2009.03.008 (PMC2707763; doi:10.1016/j.bbamcr.2009.03.008)

Supplementary Figure 1

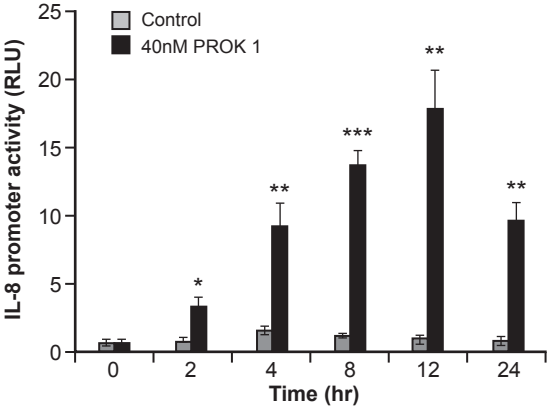

Supplement: Supplementary Fig. 1 — An IL-8 promoter-luciferase construct transfected into Ishikawa PROKR1 cells was activated by treatment with 40 nM PROK1 but not by control treatment. Data are presented as mean ± S.E. of n = 3 experiments. ⁎p < 0.05, ⁎⁎p < 0.01, ⁎⁎⁎p < 0.001. Control = vehicle treatment of Ishikawa PROKR1 cells. [file mmc1.pdf]

Supplementary figure 2

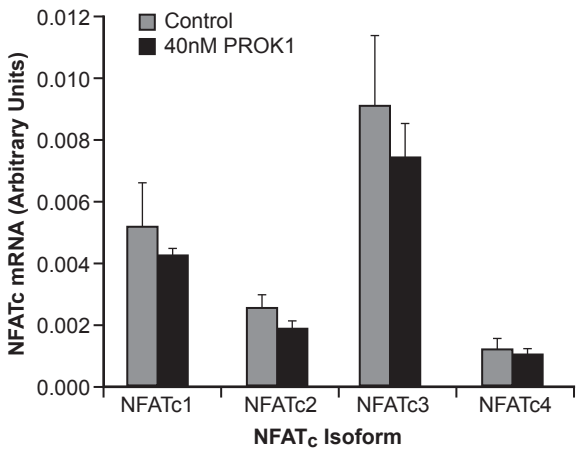

Supplement: Supplementary Fig. 2 — mRNA expression for NFAT isoforms NFATc1, NFATc2, NFATc3 and NFATc4 was detected in Ishikawa PROKR1 cells. No induction of NFATc isoforms was observed in response to treatment with 40 nM PROK1 for 8 h. Data are presented as mean ± S.E. of n = 3 experiments. [file mmc2.pdf]

Supplementary Figure 3

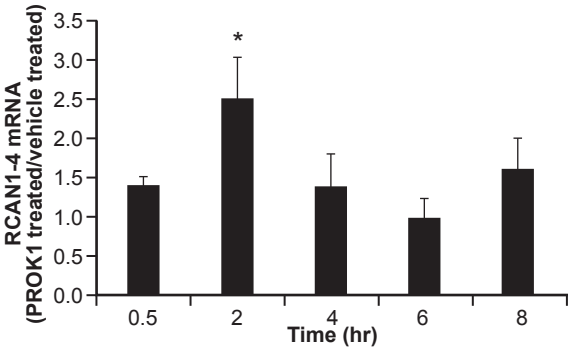

Supplement: Supplementary Fig. 3 — Human first trimester decidua treated with 40 nM PROK1 showed a significant increase in the expression of RCAN1-4 mRNA. Data are presented as mean ± S.E. of n = 7 experiments. ⁎p < 0.05. [file mmc3.pdf]

Supplementary Figure 4

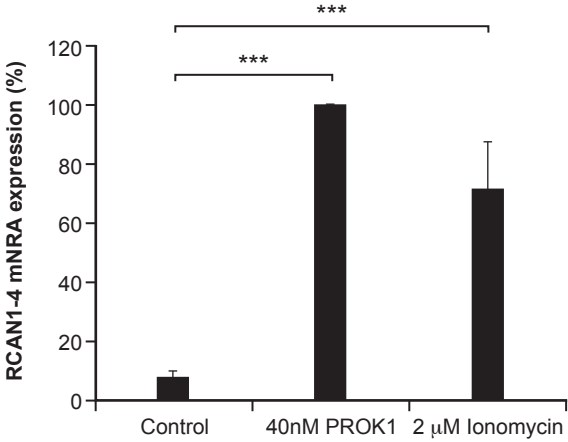

Supplement: Supplementary Fig. 4 — Treatment of Ishikawa PROKR1 cells with ionomycin (2 μM) resulted in an increase in RCAN1-4 mRNA similar to that induced by PROK1. Data is presented as percentage of mRNA expression relative to PROK1 treated cells and represent the mean ± S.E. of n = 3 experiments. ⁎⁎⁎p < 0.001. Control = vehicle treatment of Ishikawa PROKR1 cells. [file mmc4.pdf]
